# Fast and robust T1 mapping based on a 3D dual‐echo UTE sequence (PETALUTE) for superparamagnetic iron oxide nanoparticle (SPION) biodistribution assessment

**DOI:** 10.1002/mp.70675

**Published:** 2026-09-24

**Authors:** Zhen Jiang, Stephen Sawiak, Alexandra Lipka, Xin Shen, Uzay Emir, Ali Özen, Mark Chiew, Justin Geise, Joseph Speth, Deng‐Yuan Chang, Jessica Veenstra, Mitchell Gabalski, Luis Solorio, Gregory Tamer, Matthew Scarpelli

**Affiliations:** ^1^ School of Health Sciences College of Health and Human Sciences Purdue University West Lafayette Indiana USA; ^2^ Department of Physiology Development and Neuroscience University of Cambridge Cambridge UK; ^3^ Department of Clinical Neurosciences Wolfson Brain Imaging Centre Cambridge UK; ^4^ College of Engineering Purdue University West Lafayette Indiana USA; ^5^ Division of Medical Physics in Radiology German Cancer Research Center (DKFZ) Heidelberg Germany; ^6^ Department of Radiology University of California San Diego San Diego California USA; ^7^ Department of Radiology University of North Carolina at Chapel Hill Chapel Hill North Carolina USA; ^8^ Department of Radiology Medical Physics Faculty of Medicine Medical Center‐University of Freiburg Freiburg Germany; ^9^ Nuffield Department of Clinical Neurosciences University of Oxford Oxford UK; ^10^ Physical Sciences Platform Sunnybrook Research Institute Toronto Ontario Canada; ^11^ Department of Medical Biophysics University of Toronto Toronto Ontario Canada; ^12^ Weldon School of Biomedical Engineering Purdue University West Lafayette Indiana USA

**Keywords:** ferumoxytol, T1 mapping, UTE

## Abstract

**Background:**

Superparamagnetic iron oxide nanoparticles (SPIONs), such as ferumoxytol, are promising theranostic agents that can be assessed with MRI. Relaxation time mapping can provide reproducible and quantitative biomarkers of SPION distribution, suitable for longitudinal and cross‐individual studies. However, conventional approaches suffer from strong susceptibility artifacts, long echo times (TE), and prolonged scan times, which limit the accurate quantification of SPION biodistribution.

**Purpose:**

To address the limitations of conventional approaches, this study aimed to develop a fast, B_1_^+^‐corrected $T_{1}$ mapping protocol based on PETALUTE (a 3D dual‐echo ultrashort echo time MRI sequence with a petal‐like rosette k‐space trajectory) using a variable flip‐angle (VFA) acquisition for T_1_ mapping to assess ferumoxytol distribution.

**Methods:**

Agarose phantoms containing 0–5000 µg/mL ferumoxytol were scanned on a preclinical 7T MRI system using PETALUTE and RARE‐VTR (rapid acquisition with relaxation enhancement and variable repetition time). PETALUTE T_1_ maps were computed from two VFA acquisitions (4° and 20°). Mean R_1_ values were correlated with ferumoxytol concentration to assess the method's reliability. For in vivo feasibility testing, mice bearing 4T1 mammary tumors and flank tumors were assigned to the control (*n* = 1) or ferumoxytol‐injected group (*n* = 2; 40 mg/kg i.v.). Abdominal MRI scans were performed 24 h post‐injection with both PETALUTE and RARE‐VTR. Regions of interest in the thigh muscle, mammary tumors, and flank tumors were analyzed to compare the estimated T_1_ and R_1_ values obtained with both methods.

**Results:**

In the phantom study, PETALUTE preserved positive contrast for ferumoxytol at all concentrations, whereas the conventional RARE‐VTR sequence exhibited signal loss and hypointensity. For PETALUTE, there was a significant linear correlation between R_1_ and ferumoxytol concentration (*R* = 0.975, *p* < 0.01), while RARE‐VTR showed no significant correlation (*R* = 0.672, *p* = 0.144). In vivo, PETALUTE provided high‐resolution, non‐gated, whole‐abdominal images with short acquisition times (4 min 19 s). In ferumoxytol‐injected mice, flank tumors exhibited T_1_ shortening, consistent with the expected iron accumulation. The dual‐echo capabilities of PETALUTE facilitated observation of elevated T_1_ with preserved T_2_*‐weighted signal in one of the mammary tumors.

**Conclusions:**

The proposed PETALUTE‐based T_1_ mapping enables fast and positive‐contrast ferumoxytol imaging with higher spatial coverage and more stable measurements across a wider concentration range than conventional RARE‐VTR T_1_ mapping.

## 1 INTRODUCTION

Nanoparticles (NPs) are a powerful platform for precision cancer therapy, enhancing the therapeutic index by concentrating agents in tumors while minimizing off‐target exposure.^1^, ^2^ In practice, NP uptake varies widely across patients and tumor types.^3^, ^4^ This variability makes the effective dose unpredictable, complicating treatment planning and clinical translation. Theranostic NPs, which combine therapeutic and imaging functions in a single platform, have therefore been used for the simultaneous treatment and visualization of NP delivery.^5^, ^6^, ^7^ However, a noninvasive imaging modality is still required to confirm target engagement and enable personalized, response‐adaptive dosing strategies.^4^

Magnetic resonance imaging (MRI) is well‐suited for theranostic NP tracking. It is non‐ionizing, widely available, and offers high spatial resolution with excellent soft‐tissue contrast. Superparamagnetic iron oxide nanoparticles (SPIONs), such as ferumoxytol, are MRI‐visible theranostic NPs owing to their intrinsic physical properties.^8^ They shorten both the transverse (T_2_/T_2_*) and longitudinal relaxation times (T_1_)^9^, ^10^, ^11^ appearing brighter on T_1_‐weighted MRI images and darker on T_2_/T_2_*‐weighted MRI images.

However, MRI signal intensity alone is not a reliable quantitative indicator of SPION, as it is inherently arbitrary. Quantitative MRI (qMRI) is a more reliable approach that offers reproducible measurements grounded in physics models of the MR signal, derived from multiple images acquired with varied parameters to capture tissue‐specific properties.^12^ In particular, T_1_ and T_2_/T_2_* mapping yield voxel‐wise parametric maps that encode quantitative relaxation values, reflecting tissue composition and the local magnetic environment.^13^, ^14^, ^15^, ^16^ These relaxation mapping techniques have also been investigated for assessing SPION biodistribution^9^, ^17^, ^18^, ^19^, ^20^, ^21^ although several challenges remain in their application to SPION imaging.

T_2_/T_2_* mapping is sensitive to SPIONs, but its accuracy and efficiency decline as concentration increases.^9^ At high concentrations, strong susceptibility artifacts cause the blooming effect and signal voids, degrading spatial localization and measurement accuracy.^20^ In contrast, T_1_ mapping is more robust to susceptibility artifacts and can offer a higher signal‐to‐noise ratio (SNR) than T_2_ mapping,^20^ but it still has essential limitations in SPION imaging. First, conventional T_1_ mapping methods, such as Inversion Recovery Spin‐Echo (IR‐SE) and Rapid Acquisition with Relaxation Enhancement with Variable TR (RARE‐VTR), utilize echo times (TE) longer than the ultrashort T_2_ of SPION‐rich regions, resulting in T_2_‐related signal loss and inaccurate T_1_ calculations. In addition, a long repetition time (TR) is usually required to achieve complete relaxation for T_1_ mapping, increasing susceptibility to motion artifacts and hindering clinical feasibility.

Ultra‐short echo time (UTE) MRI is a promising technique to address the challenges described above.^21^, ^22^ It can capture rapidly decaying signals and provide positive T_1_ contrast for SPIONs.^23^ UTE can also be combined with a variable‐flip‐angle (VFA) approach to enable rapid T_1_ mapping for SPIONs assessment.^24^ However, the UTE MRI signal can be challenging to interpret in vivo because the short‐T_2_* signal from SPION is often masked by the long‐T_2_* signal from water and fat (e.g., necrotic tissue)^22^, ^25^ or confounded by other short‐T_2_* components such as calcification or hemorrhagic byproducts.^26^ Dual‐echo UTE methods have been developed to separate short‐ and long‐T_2_* components, but, to our knowledge, their applications have mostly been tissue‐specific rather than focused on SPION tracking.^27^, ^28^ A dual‐echo UTE T_1_ mapping approach needs to be adapted for SPION monitoring.

**FIGURE 1:**
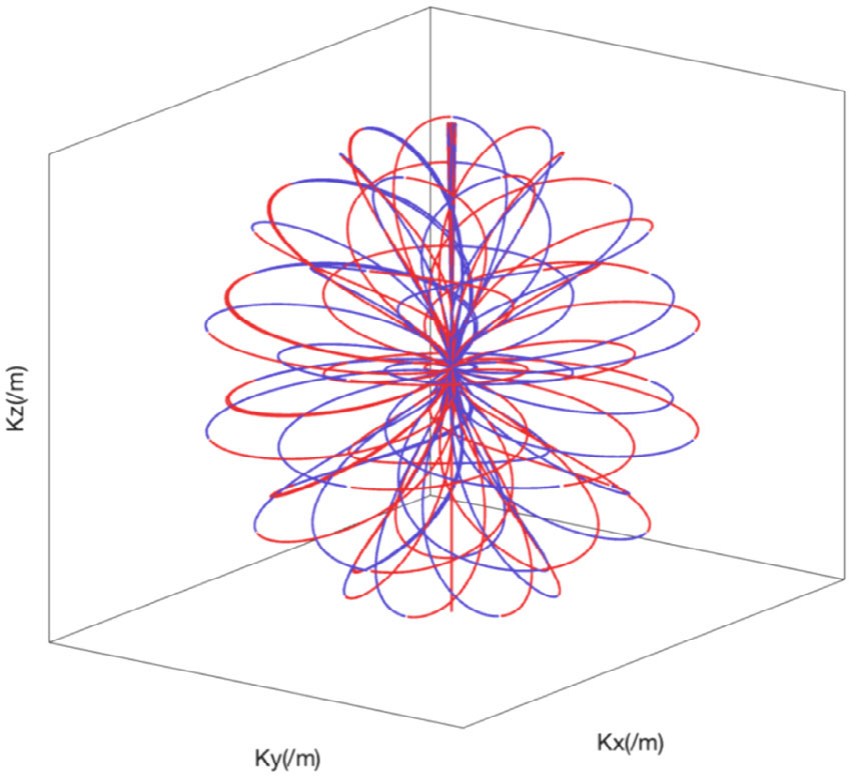
3D rosette k‐space trajectory used in PETALUTE. The sampling path traces petal‐like loops in k‐space, repeatedly passing through the center while covering peripheral regions in multiple directions. Blue and red curves indicate the first and second echoes, respectively.

To achieve this, we propose a T_1_ mapping protocol based on PETALUTE, a 3D dual‐echo UTE MRI sequence with a rosette k‐space trajectory, in which the sampling path traces repeated petal‐like loops in k‐space (Figure 1).^29^, ^30^ Compared with conventional UTE‐VFA methods, PETALUTE combines dual‐echo acquisition with efficient volumetric sampling, enabling collection of both ultrashort‐TE and longer‐TE signals within a single acquisition. This combination is particularly advantageous for SPION imaging because it improves sensitivity to SPION‐related signal while providing longer‐TE information that may help distinguish SPION‐related signal from long‐T_2_* components. In addition, the rosette k‐space trajectory supports high spatial resolution with broad volumetric coverage, enhancing the practicality.

In this study, we developed and evaluated a PETALUTE T_1_ mapping protocol that combines VFA acquisition^31^ and B_1_^+^ map correction^32^ for ferumoxytol assessment in vitro and in vivo. We hypothesize that PETALUTE will maintain a positive contrast of ferumoxytol while reducing susceptibility artifacts, even at high concentrations, and will enable acquisition of two contrasts with different TEs. To test this hypothesis, we compared PETALUTE T_1_ maps with conventional RARE‐VTR T_1_ maps across a range of ferumoxytol concentrations in phantoms. In addition, we assessed the in vivo feasibility of using PETALUTE T_1_ maps for evaluating ferumoxytol in a dual‐tumor mouse model.

## 2 MATERIALS AND METHODS

### 2.1 T1 map calculation

**FIGURE 2:**
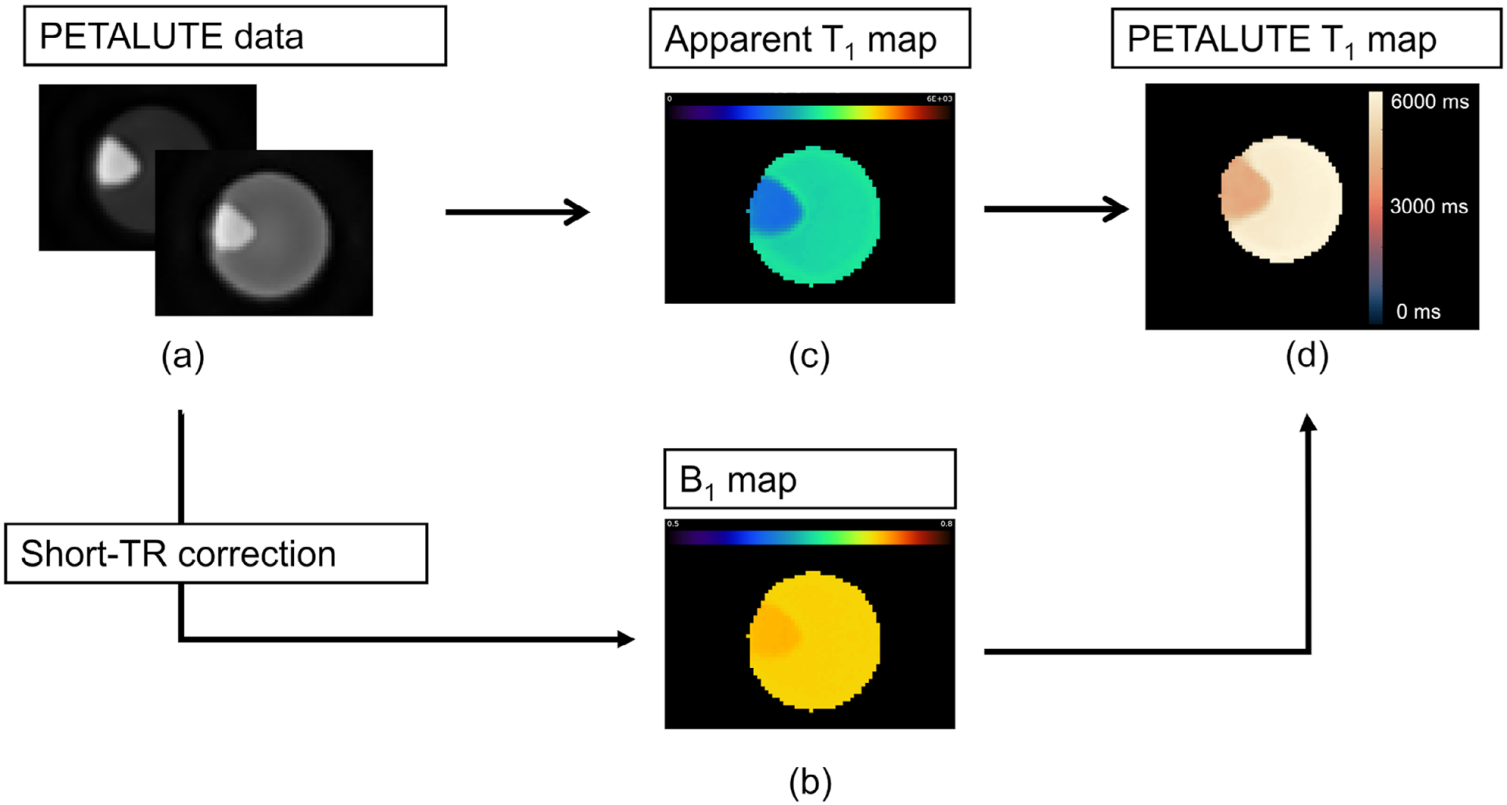
PETALUTE T1 mapping workflow. (a) PETALUTE image reconstructed using nonuniform fast Fourier transform method. (b) B1+ map generated by the variable flip angle method with short‐TR correction and smoothed using a Gaussian filter with σ = 3; color bar range from 0.5 to 0.8. (c) Apparent T1 map generated using the two flip‐angle PETALUTE images; color bar range from 0 to 6000 ms. (d) PETALUTE T1 map derived from the apparent T1 map corrected using the B1+ map; color bar range from 0 to 6000 ms.

PETALUTE T_1_ mapping workflow is illustrated in Figure 2. We first generated apparent T_1_ maps using PETALUTE first echoes from the VFA acquisitions (4°, i.e., the Ernst angle, and 20°) using Equation 1, based on the spoiled gradient‐echo signal equation.^31^, ^33^

(1) $$T_{1,\mathrm{app}}=2\;\mathrm{TR}\frac{S_{1}/\alpha_{1}-S_{2}/\alpha_{2}}{S_{2}\cdot \alpha_{2}\;-S_{1}\cdot \alpha_{1}}$$

T_1, app_, represents the apparent T_1_ value; and S_1_, S_2_ are signal intensities from PETALUTE first echoes acquired with flip angles of 4° (ɑ_1_) and 20° (ɑ_2_), respectively.

(2) $$\begin{array}{cc} r & =\frac{S_{1,\infty }}{S_{2,\infty }}=\frac{\sin\alpha_{1}}{\sin\alpha_{2}}=\frac{\sin\alpha_{1,\mathrm{act}}}{\sin5\alpha_{1,\mathrm{act}}}\\ & =\frac{\sin\alpha_{1,\mathrm{act}}}{5\sin\alpha_{1,\mathrm{act}}-20\mathrm{si}n^{3}\alpha_{1,\mathrm{act}}+16\mathrm{si}n^{5}\alpha_{1,\mathrm{act}}} \end{array}$$

Under a fully relaxed condition (TR ≥5T_1_), the ratio between the two signal intensities, r, can be expressed as in Equation 2,^34^ where ɑ_1, act_ was the actual flip angle.

The PETALUTE sequence, however, uses a very short TR that prevents complete longitudinal relaxation. Therefore, a short‐TR correction was applied to estimate the fully relaxed signals.^32^

The actual flip angle ɑ_1, act_ was then derived from Equation 3:

(3) $$\alpha_{1,\mathrm{act}}=\arcsin\left(\frac{\sqrt{5-\sqrt{5+\frac{4}{r}}}}{8}\right)$$

(4) $$B_{1}^{+}(x,\;y,\;z)=\alpha_{1,\mathrm{act}}\;(x,\;y,\;z)/\alpha_{1}$$

The corresponding voxel‐wise B_1_^+^ map was then computed as the ratio of the actual flip angle to nominal flip angle (Equation 4).

Finally, the B_1_^+^ map was applied to correct the apparent T_1_ map, producing the PETALUTE T_1_ map (Equation 5).

(5) $$T_{1}(x,\;y,\;z)=T_{1,\mathrm{app}}\;(x,\;y,\;z)\cdot {\left(B_{1}^{+}\left(x,\;y,\;z\right)\right)}^{-2}$$

(6) $$S(\mathrm{TR})=S_{0}\;(1-e^{-\mathrm{TR}/T_{1}})$$

The RARE‐VTR T_1_ maps were computed by voxel‐wise nonlinear least‐squares fitting of a mono‐exponential recovery model (Equation 6) to the multi‐TR data.

### 2.2 Imaging protocol and reconstruction

Imaging protocol included vendor‐provided RARE‐VTR, Multi‐Echo Gradient‐Echo (MGE) and the proposed PETALUTE sequences. For the in vivo feasibility study, the MGE sequence was included to provide a reference T_2_* weighted image for assessment of tissue contrast and quantitative T_2_* analysis was not performed in this study. All imaging protocol parameters are summarized in Table 1. For PETALUTE, the reported acquisition time corresponds to a single flip‐angle acquisition. Since two flip angles were acquired for T_1_ estimation, the total acquisition time for one PETALUTE T_1_ map was 2 min 10 s in the phantom study and 8 min 38 s in the in vivo feasibility study. For RARE‐VTR and MGE, the reported acquisition time corresponds to the full multi‐TR protocol used for T_1_ estimation. All scans were performed on a 7T horizontal‐bore small animal MRI system (BioSpec 70/30; Bruker Instruments; maximum gradient strength: 660 mT/m; maximum slew rate: 4570 T/m/s).

**TABLE 1:** Image protocol parameters for RARE‐VTR, MGE, and PETALUTE sequences used in phantom and in vivo acquisitions.

	RARE‐VTR^a^	MGE^b^	PETALUTE^c^
Resolution	0.234 × 0.234 × 1 mm^3^	0.234 × 0.234 × 1 mm^3^	0.2 × 0.2 × 0.2 mm^3^
FOV^d^	30 × 30 mm^3^ 20 slices (thickness = 1 mm)	30 × 30 mm^3^ 20 slices (thickness = 1 mm)	40 × 40 × 40 mm^3^ (Phantom); 60 × 60 × 60 mm^3^ (In‐vivo)
TE^e^	20.3 ms	2 ms, 6 ms, 10 ms, 14 ms, 18 ms, 22 ms, 26 ms, 30 ms, 34 ms, 38 ms	0.016 ms (first echo) 3.2 ms (second echo)
TR^f^	934.58 ms, 1788.44 ms, 2986.55 ms, 5011.78 ms, 15000.0 ms	1500 ms	7 ms
Flip angle	90°	30°	4°, 20°
Acquisition time	6 min 11 s	6 min 24 s	1 min 5 s/flip‐angle (Phantom) 4 min 19 s/flip‐angle (In‐vivo)

^a^ RARE‐VTR = rapid acquisition with relaxation enhancement–variable TR.

^b^ MGE = multi‐gradient echo.

^c^ PETALUTE = 3D dual‐echo ultrashort echo time.

^d^ FOV = field of view.

^e^ TE = echo time.

^f^ TR = repetition time.

RARE‐VTR and MGE images were reconstructed on‐scanner automatically using Bruker ParaVision 6.0.1. PETALUTE data were processed in MATLAB (202Xa, MathWorks, USA). The nonuniform fast Fourier transform (NUFFT) method was used to reconstruct the PETALUTE images using BART (Berkeley Advanced Reconstruction Toolbox; nufft for NUFFT reconstruction, ecalib for ESPIRiT coil‐sensitivity estimation, and pics for iterative CS‐SENSE) and MIRT (Michigan Image Reconstruction Toolbox; Gmri and ir_mri_density_comp to define the non‐Cartesian encoding operator and density‐compensation weights).

### 2.3 Phantom study

Seven phantoms at nominal ferumoxytol concentrations of 0, 100, 250, 750, 1000, 1250, and 5000 µg/mL were prepared using 4.2% agarose (w/v) and ferumoxytol (Feraheme, AMAG Pharmaceuticals). Ferumoxytol was mixed into the heated agarose solution under continuous stirring. The ferumoxytol‐agarose mixture was allowed to cool at room temperature until gelation. After solidification, a small piece of the ferumoxytol‐containing agarose was removed and coated with a thin layer of water‐resistant polyurethane adhesive to maintain its integrity. This piece was then embedded in a 1 mL microcentrifuge tube prefilled with 4.2% agarose (w/v) to form the final phantom. This design produced a compact phantom that remained compatible with the size constraints of the MRI coil, while the ferumoxytol‐containing agarose exhibited positive contrast relative to the surrounding agarose. After cooling to room temperature, all the phantoms were stored in a refrigerated environment. They were allowed to warm at room temperature for two hours before scanning.

**FIGURE 3:**
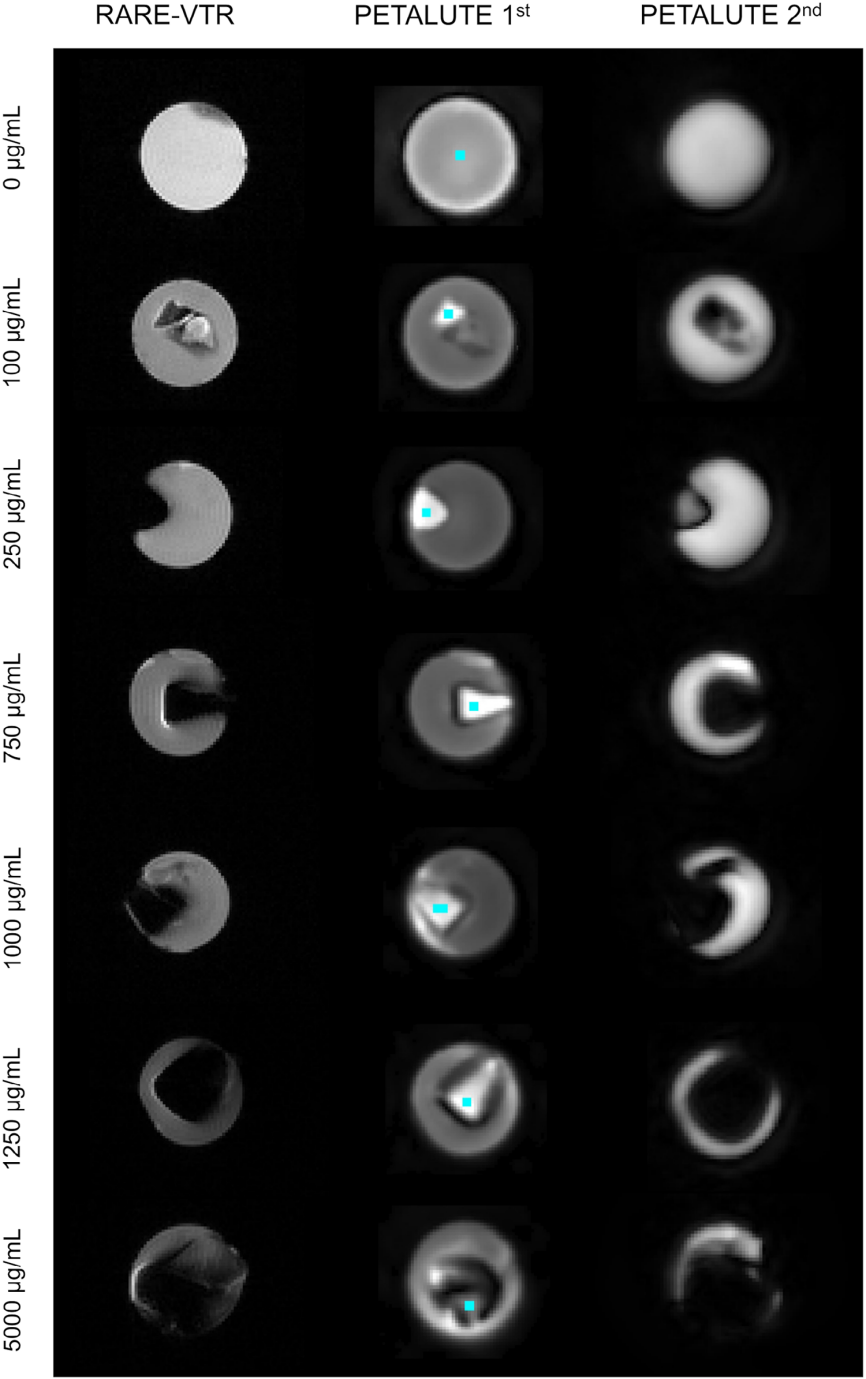
Reconstructed images of phantoms using RARE‐VTR and PETALUTE. Phantoms containing various concentrations of ferumoxytol (0 – 5000 ppm) appear as bright regions in PETALUTE first echo (flip angle = 4°) and dark regions in PETALUTE second echo (flip angle = 4°) and RARE‐VTR images (TR = 934.58 ms). The ROI is outlined in blue.

All phantoms were scanned using a 23‐mm‐diameter Tx/Rx 1H RF volume coil (Bruker BioSpin, Ettlingen, Germany). PETALUTE images were acquired with a total of 9,216 petals, each petal containing 206 sampling points with 103 sampling points per echo. RARE‐VTR and PETALUTE T_1_ maps were calculated as previously described. Regions of interest (ROIs) were defined in 3D Slicer^35^ around the ferumoxytol (Figure 3). A fixed ROI size of 45 voxels (0.36 mm^3^) was used for all phantoms to maintain consistency. For each concentration, the mean and standard deviation of T_1_ and R_1_ were calculated within the ROI.

The relaxation rate R_1_, defined as the reciprocal of T_1_, has an approximately linear relationship with the concentration of ferumoxytol, as described in Equation 7.^19^

(7) $$R_{1}\;\left(C\right)=R_{1,0}+a\cdot C$$

where C is the ferumoxytol concentration (µg/mL), R_1, 0_ is the intrinsic R_1_ of the agarose solution (s^−1^), and a is the slope (mL·µg^−1^·s^−1^).

The calibration curve was established by linear regression analysis of R_1_ versus ferumoxytol concentration up to 1250 µg/mL. A 5000 µg/mL ferumoxytol phantom was included to assess the upper practical limit of the current experimental setup.

### 2.4 In vivo feasibility study

Female BALB/c mice (8–10 weeks old) were obtained from Envigo (Lafayette, Indiana) and housed in the local facility under pathogen‐free conditions. All experimental procedures were reviewed and approved by the local institution in accordance with ARRIVE guidelines.

4T1 cells were obtained from American Type Culture Collection (ATCC) and cultured in RPMI‐1640 medium containing 10% FBS and 1% penicillin/streptomycin. All cultures were incubated at 37°C under humidified conditions with 5% CO_2_ and 95% air.

Three female BALB/c mice bearing 4T1 mammary and flank tumors (dual tumor model) were used in this experiment. To establish the model, mice were subcutaneously injected with a single‐cell suspension of 1 × 10^5^ 4T1 tumor cells in 50 µL of phosphate‐buffered saline (PBS) into the fourth right mammary fat (designated as primary tumor), and 5 × 10^4^ 4T1 cells in 50 µL of PBS into the left upper thigh (designated as flank tumor). Mice were divided into control (*n* = 1) and ferumoxytol‐injected (*n* = 2) groups. Fourteen days after tumor implantation, the ferumoxytol group received a single dose of 40 mg/kg ferumoxytol administered via the tail vein.

MRI scans were conducted 24 h after injection. All mice were scanned using a 7T MRI system with a 40‐mm‐diameter Tx/Rx 1H RF volume coil (Bruker BioSpin, Ettlingen, Germany), and anesthesia was maintained at 2% isoflurane in oxygen throughout the imaging process. PETALUTE images were acquired using 36,864 petals with 206 sampling points per petal (103 sampling points per echo). RARE‐VTR images were also obtained for comparison.

ROIs were manually delineated in the thigh muscle (as a reference region), mammary tumors, and flank tumors using 3D Slicer.^35^ For the thigh muscle reference region, the ROI was placed in the mid‐thigh to avoid the adjacent bone, and intramuscular vessels were excluded from analysis to minimize vascular partial‐volume effects (Figure 6). Quantitative analyses were performed in MATLAB. For each ROI, the mean and standard deviation of T_1_ and R_1_ were calculated from both PETALUTE and RARE‐VTR T_1_ maps to enable comparative evaluation.

## 3 RESULT

### 3.1 Phantom study

Figure 3 presents reconstructed images of all phantoms acquired using RARE‐VTR and PETALUTE, demonstrating the positive contrast of ferumoxytol produced by the PETALUTE first echo and the negative contrast by the second echo. It should be noted that the 5000 µg/mL phantom showed degraded ferumoxytol‐to‐agarose contrast and reduced reliability of PETALUTE R_1_ quantification at this concentration (Tables S1–S3 and Figures S2, S3). In contrast, the RARE‐VTR images showed signal loss and hypointensity at every tested ferumoxytol concentration.

**FIGURE 4:**
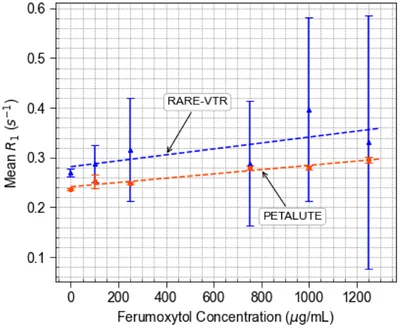
Linear regression of mean R1 versus ferumoxytol concentration for PETALUTE (circles) and RARE‐VTR (triangles). Error bars represent the standard deviation within each ROI. The R values were 0.975 for PETALUTE and 0.672 for RARE‐VTR.

To evaluate the concentration‐dependent R_1_ response, the relationship between mean R_1_ values and ferumoxytol concentration was analyzed (Figure 4). A significant positive linear correlation was observed for PETALUTE‐based mean R_1_ (*R* = 0.975, *p* < 0.01). In contrast, the RARE‐VTR‐based mean R_1_ did not show significant correlation (*R* = 0.672, *p* = 0.144). Additionally, PETALUTE‐based R_1_ showed lower standard deviations (0.002–0.006 s^−^^1^) than those of RARE‐VTR (0.008–0.255 s^−^^1^) across the full concentration range, indicating improved precision.

### 3.2 In vivo feasibility study

**FIGURE 5:**
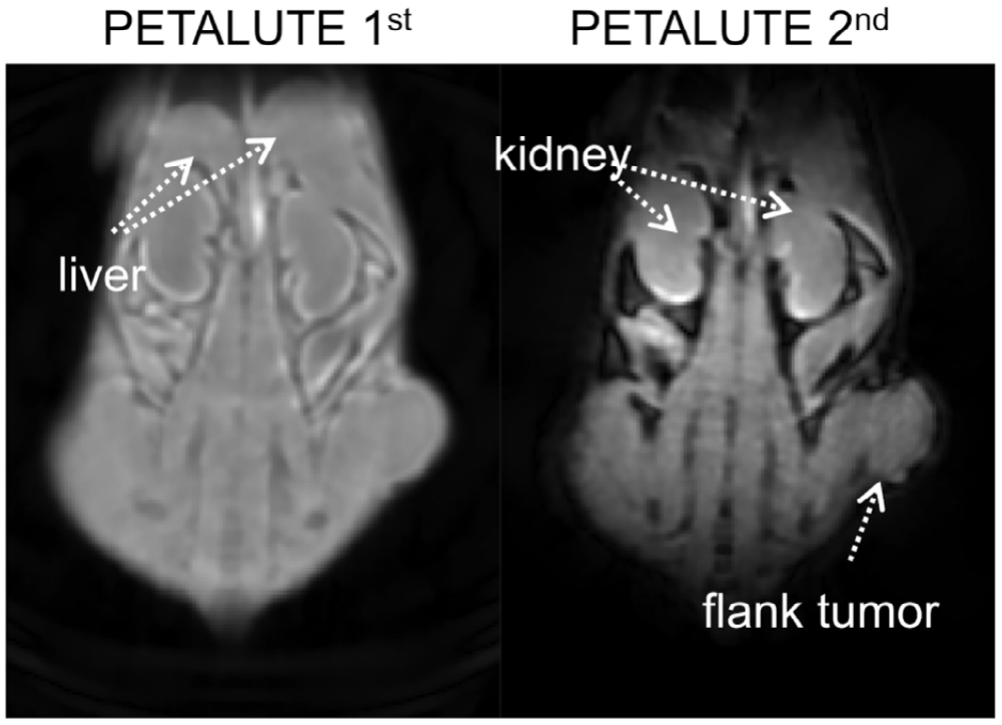
Representative coronal reformatted PETALUTE images from a control mouse (Mouse 47) showing the first echo and second echo (FA = 4°). The figure illustrates the full abdominal coverage by the PETALUTE acquisition.

**FIGURE 6:**
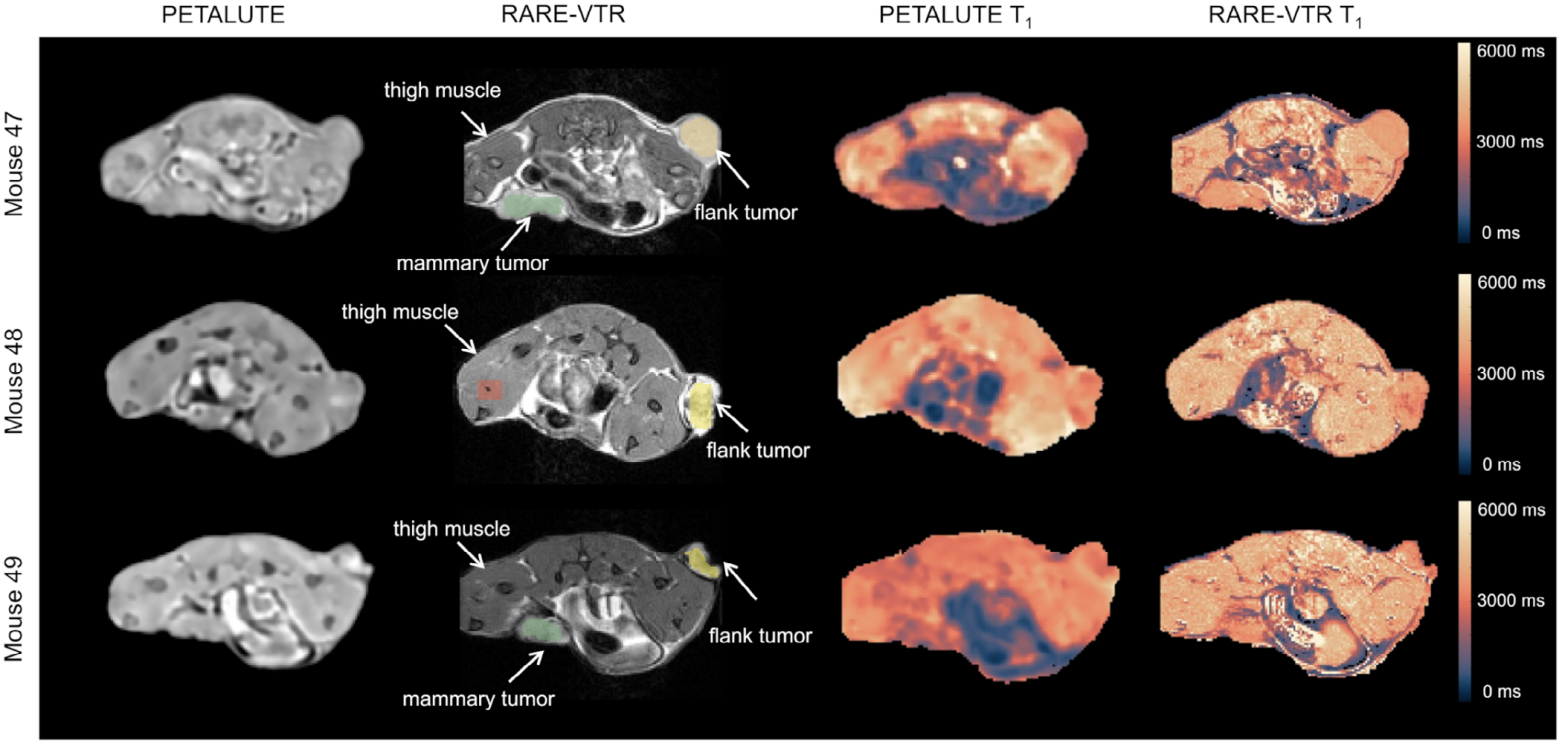
PETALUTE and RARE‐VTR images and corresponding T_1_ maps from the control mouse (Mouse 47) and the ferumoxytol‐injected mice (Mouse 48 and Mouse 49). PETALUTE images are first echo acquired with a flip angle of 4°, while RARE‐VTR images are acquired with TR = 934.58 ms. ROIs used for quantitative analysis are overlaid on the RARE‐VTR anatomical images: thigh muscle (red), flank tumor (yellow), and mammary tumor (green). The thigh muscle ROI for Mouse 47 and Mouse 49 and the mammary tumor ROI for Mouse 48 were located on different slices and are therefore not shown. Color maps show T_1_ values in milliseconds, with a scale range of 0–6000 ms.

Reconstructed PETALUTE images covered the whole abdominal region and provided visualization of anatomical features (Figure 5). Notably, structures such as the kidneys and livers, which were poorly defined in the RARE‐VTR images due to a limited field of view, were distinguishable on PETALUTE images. In addition, while RARE‐VTR images displayed a hypointense region within the flank tumor, PETALUTE showed slightly higher signal intensity near the tumor center (Figure 6).

ROIs within the thigh muscle, flank tumors, and mammary tumors allowed the determination of T_1_ values from PETALUTE and RARE‐VTR. The overall mean T_1_ and R_1_ values for each mouse and the respective ROI are reported in Table 2. For both T_1_ mapping methods, muscle tissue mean T_1_ values remained relatively stable across mice. For RARE‐VTR, the mean muscle T_1_ values for the ferumoxytol‐injected mice were within 6% of the Control mouse T_1_ values. For PETALUTE, the mean muscle T_1_ values for ferumoxytol‐injected mice were within 4% of the Control mouse T_1_ values. In the flank tumor, both T_1_ mapping methods yielded lower mean T_1_ values in ferumoxytol‐injected mice than in the Control mouse. The T_1_ reduction in the flank tumor in ferumoxytol‐injected mice was approximately 10%–12% with RARE‐VTR and 4%–7% with PETALUTE. In the mammary tumor, T_1_ changes differed across the two T_1_ mapping methods. With RARE‐VTR, both the ferumoxytol‐injected mice exhibited an approximately 9%–11% T_1_ reduction relative to the Control mouse. PETALUTE, however, revealed divergent responses: relative to the Control mouse, the mean T_1_ increased by approximately 42% in FMX‐1 but decreased by approximately 4% in FMX‐2.

**TABLE 2:** Mean and standard deviation (SD) of T1 and R1 values within the ROIs of thigh muscle, flank tumors and mammary tumors in three mice.

ID^a^	ROI	RARE‐VTR^b^ R1 mean ± SD (s‐1)	PETALUTE^c^ R1 mean ± SD (s‐1)	RARE‐VTR T1 mean ± SD (ms)	PETALUTE T1 mean ± SD (ms)
Control	Thigh Muscle	0.242 ± 0.023	0.247 ± 0.015	4164 ± 392	4063 ± 249
	Flank Tumor	0.260 ± 0.012	0.298 ± 0.043	3850 ± 184	3424 ± 464
	Mammary Tumor	0.265 ± 0.029	0.374 ± 0.063	3821 ± 396	2745 ± 443
FMX‐1	Thigh Muscle	0.258 ± 0.029	0.240 ± 0.021	3919 ± 427	4203 ± 357
	Flank Tumor	0.295 ± 0.037	0.318 ± 0.042	3438 ± 389	3198 ± 416
	Mammary Tumor	0.302 ± 0.073	0.265 ± 0.048	3472 ± 691	3894 ± 667
FMX‐2	Thigh Muscle	0.239 ± 0.029	0.251 ± 0.020	4252 ± 545	4002 ± 322
	Flank Tumor	0.306 ± 0.060	0.307 ± 0.030	3369 ± 556	3286 ± 341
	Mammary Tumor	0.302 ± 0.060	0.386 ± 0.049	3401 ± 500	2632 ± 330

^a^ Control = control mouse; FMX‐1 and FMX‐2 = ferumoxytol‐injected mice.

^b^ RARE‐VTR = rapid acquisition with relaxation enhancement–variable TR.

^c^ PETALUTE = 3D dual‐echo ultrashort echo time.

## 4 DISCUSSION

We developed a B_1_^+^‐corrected PETALUTE‐VFA T_1_ mapping protocol that enables high‐resolution T_1_ mapping for ferumoxytol biodistribution assessment. This new protocol improved the linearity between R_1_ and ferumoxytol concentration, produced positive contrast at high ferumoxytol concentrations, and expanded spatial coverage, thereby overcoming the poor image quality and signal losses that limit conventional T_1_ mapping for SPION imaging.

PETALUTE T_1_ mapping establishes a reliable calibration curve for ferumoxytol assessment up to 1250 µg/mL, whereas the conventional RARE‐VTR method did not yield a comparable linear response over this range. The improved performance of PETALUTE T_1_ mapping in the phantom study is attributed to its ultrashort echo time, which captures signal from iron before substantial T_2_*‐related dephasing and therefore yields higher SNR and reduced variability in estimated T_1_ values. Consistent with this, the PETALUTE‐based R_1_ standard deviation remained relatively low across the concentration range, whereas the RARE‐VTR‐based R_1_ standard deviation increased substantially with ferumoxytol concentration (Figure 4), reaching approximately 40 times that of PETALUTE at 1250 µg/mL. This smaller concentration‐dependent variability of PETALUTE‐based R_1_ further supports the lower susceptibility of PETALUTE to T_2_*‐related signal loss at high ferumoxytol concentrations. In addition, reduced susceptibility effects at lower magnetic field strengths are anticipated to extend the usable linear range of the calibration curve, suggesting that the limits reported here at 7T are likely conservative compared with low‐field ferumoxytol assessment methods.^36^

Another factor affecting comparability between PETALUTE and RARE‐VTR is spatial resolution. PETALUTE provides high isotropic resolution, whereas RARE‐VTR is anisotropic (Table 1), particularly because of its thicker slice dimension. The larger voxel volume in RARE‐VTR increases susceptibility to partial‐volume effects, such that the measured T_1_ may reflect an average of heterogeneous ferumoxytol concentrations within a single voxel. In contrast, the isotropic resolution of PETALUTE reduces partial‐volume averaging and improves spatial specificity of the measured ferumoxytol distribution. Therefore, differences between PETALUTE and RARE‐VTR measurements may reflect not only sequence‐specific T_1_ estimation behavior, but also differing sensitivity to partial‐volume effects.

A discrepancy of about 12% was observed in the baseline agarose phantom R_1_ values (0 µg/mL ferumoxytol) between the two methods (RARE‐VTR: 0.270 s^−^^1^; PETALUTE: 0.237 s^−^^1^). This difference likely comes from systematic bias caused by different T_1_ mapping methods. RARE‐VTR and PETALUTE use different acquisition strategies, signal models, and fitting methods. These differences can lead to offsets in the measured R_1_ values. The discrepancy may also come from residual bias in the short‐TR VFA framework, including imperfect B_1_^+^ correction and incomplete spoiling.^37^ Spatial resolution may also play a role, but partial‐volume effects are less likely to be a major cause in this relatively homogeneous phantom. Similar systematic differences between VFA‐based T_1_ mapping and reference relaxation‐based methods have been reported previously.^38^ Despite this baseline offset, PETALUTE still showed excellent linearity up to 1250 µg/mL. Notably, the Bland‐Altman analysis indicates a residual proportional bias between the PETALUTE and RARE‐VTR methods after B_1_^+^ correction (Figure S4). Therefore, PETALUTE and RARE‐VTR T_1_ mapping should not be considered interchangeable quantitative T_1_ methods at this stage. Rather, PETALUTE is more suitable for relative or trend‐based assessment of ferumoxytol‐induced T_1_ change.

The linear R_1_ behavior observed in homogeneous phantoms does not establish quantitative validity in the biologically heterogeneous environment of in vivo tissue. Unlike the controlled phantom environment, in vivo tissue contains variable water content, necrosis, inflammation, hemorrhage, and spatially heterogeneous ferumoxytol distribution, all of which can influence the measured T_1_. Therefore, in vivo T_1_ should be interpreted as a ferumoxytol‐sensitive marker rather than a direct concentration readout. Nevertheless, the phantom calibration curve provides a useful reference for the expected relationship between R_1_ change and ferumoxytol accumulation, enabling relative quantitative assessment when comparing baseline and post‐injection R_1_ values.^39^

The in vivo scanning demonstrated the feasibility of using the PETALUTE T_1_ mapping protocol to assess ferumoxytol distribution in rodent tumors. Given that no substantial accumulation of ferumoxytol was expected in muscle tissue, the muscle served as a reference region. As expected, we observed minimal differences in T_1_ values in this region between the control and ferumoxytol‐injected groups, with mean muscle T_1_ values within 6% for RARE‐VTR and within 4% for PETALUTE relative to the Control mouse. In tumors, accumulation of ferumoxytol would be expected to lead to T_1_ shortening, including decreased T_1_ values and increased T_1_‐weighted MRI signal intensity. This behavior was observed in the flank tumors of ferumoxytol‐injected mice in our study, which exhibited lower T_1_ values with both methods and higher intratumoral signal intensity in the PETALUTE first echo. This finding is consistent with the preferential accumulation of ferumoxytol within tumor tissue, consistent with prior reports of tumor‐specific ferumoxytol uptake that manifests as decreased T_1_ values.^40^ The greater T_1_ reduction observed with RARE‐VTR is likely due to its longer TE (20 ms), which leads to substantial T_2_ dephasing in the presence of ferumoxytol. This residual T_2_ weighting may reduce the effective MRI signal in RARE‐VTR images, causing hypointensity and exaggerating the apparent T_1_‐shortening effect. In contrast, the ultrashort TE of PETALUTE (0.016 ms) minimizes T_2_* decay, enabling acquisition of a more direct T_1_‐weighted signal before substantial dephasing occurs.

**FIGURE 7:**
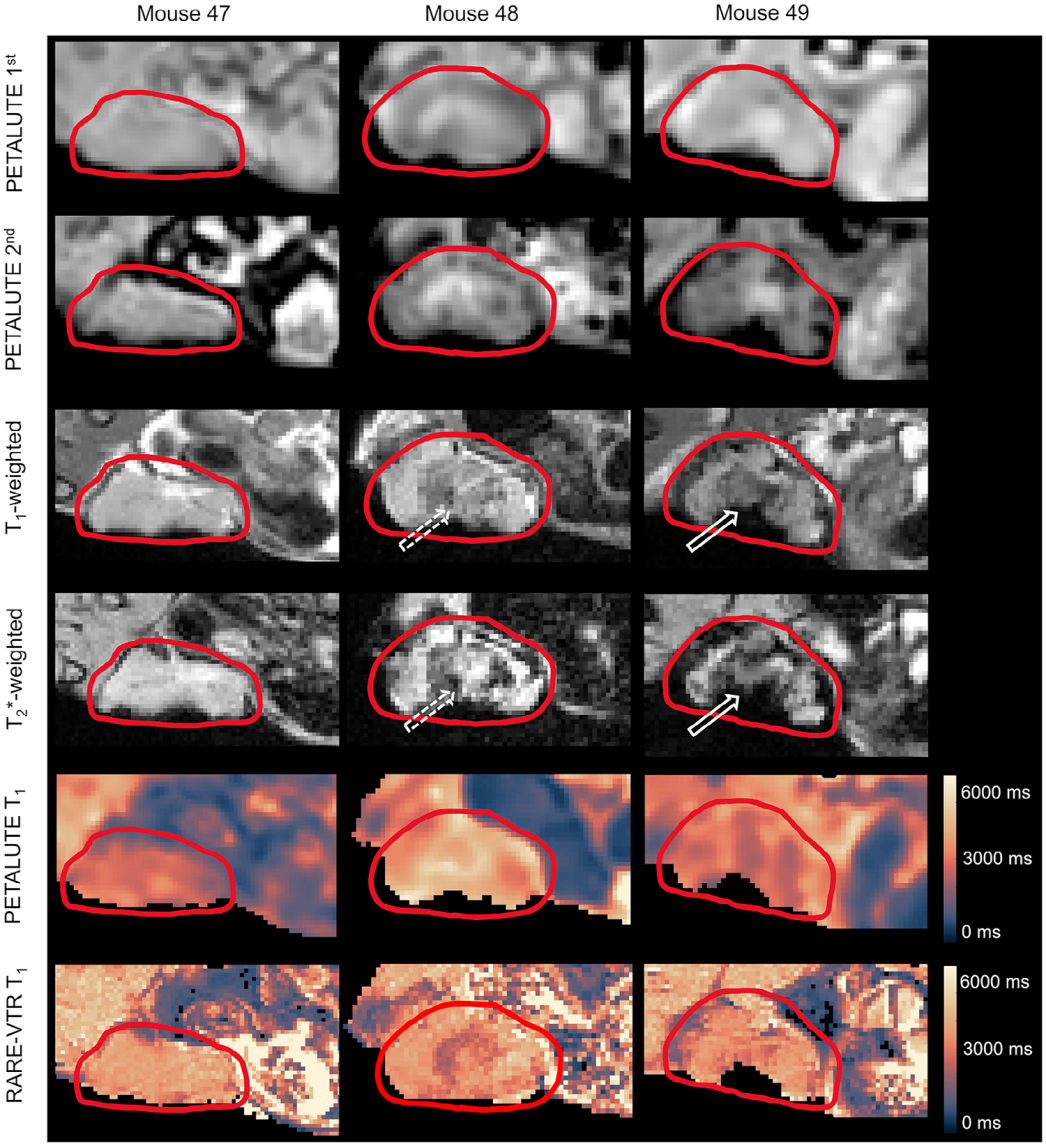
Multimodal images of the control mouse (Mouse 47) and the ferumoxytol‐injected mice (Mouse 48 and Mouse 49) focusing on the mammary tumor. Shown are PETALUTE first echo, PETALUTE second echo, T1‐weighted (RARE‐VTR, TE = 20 ms, TR = 933 ms), T2*‐weighted (MGE, TE = 6 ms, TR = 1500 ms), PETALUTE T1 map, and RARE‐VTR T1 map images. Mammary tumors are contoured in red. Dashed arrows indicate T1 hypointensity with persistent T2* signal indicated in Mouse 48 while solid arrows indicate T1 hypointensity with T2* signal loss in Mouse 49. Mouse 47 (control) showed uniform intratumor intensity. Color maps display T1 values in milliseconds, with a scale range of 0–6000 ms.

An unexpected result was observed in the in vivo study. One of the ferumoxytol‐injected mice (FMX‐1) exhibited a nearly 42% increase in mean PETALUTE T_1_ in the mammary tumor. To investigate this unexpected finding, multimodal images were examined (Figure 7). In the FMX‐1 mammary tumor, the combination of hypointensity in the T_1_‐weighted image (indicated by solid arrow in Figure 7) and persistent signal in the T_2_*‐weighted image (indicated by dashed arrow in Figure 7) is suggestive of necrosis‐like change based on prior reports.^41^ However, this interpretation remains provisional. Similar imaging features may also arise from other biological processes, including inflammation,^42^ hemorrhage,^43^ or other long‐T_2_* components. Importantly, the larger tumor volume in the FMX‐1 mammary tumor (Control: 152 mm3, FMX‐1: 210 mm3, FMX‐2: 189 mm^3^) may have increased the likelihood of necrosis, which dominated the MR signal response.^41^

Based on the phantom study (Figure 3), ferumoxytol accumulation would be expected to produce hyperintensity on the PETALUTE first echo and hypointensity on the second echo. Therefore, the hyperintensity region observed in the PETALUTE second echo of FMX‐1 (Figure 7) is not consistent with ferumoxytol accumulation alone and is more plausibly attributed to a long T_2_ component. Overall, these observations suggest that the hyperintensity observed in the FMX‐1 tumor across both PETALUTE echoes may reflect a mixture of ferumoxytol‐related signal and tissue changes. The resulting PETALUTE T_1_ map revealed elevated T_1_ values in these regions, whereas the RARE‐VTR T_1_ map was not sensitive to this effect (Figure 7). Similar intratumoral hyperintensity was also observed in both PETALUTE echoes of FMX‐2, though without measurable T_1_ elevation. This may reflect mild or early‐stage tissue alteration that was insufficient to alter the overall MR signal behavior.

The different behavior between mammary and flank tumors may also reflect systematic biological differences related to tumor location. Mammary tumors and flank tumors differ in vascularization, immune‐cell infiltration, and necrotic tendency, all of which may influence ferumoxytol delivery, retention, and the resulting T_1_ behavior. In the current setting, PETALUTE may be particularly sensitive to intratumoral biological heterogeneity. This sensitivity could be advantageous if it reflects biologically relevant variation, but it also complicates the interpretation of ferumoxytol‐related T_1_ alteration in this small exploratory cohort. Further histological studies will be required to determine whether these findings correspond to necrosis, inflammation, hemorrhage, or a combination of these processes. Given the small sample size, the mammary tumor finding in FMX‐1 should be interpreted cautiously as an individual response rather than evidence of a group‐level response.

A practical difference between PETALUTE and RARE‐VTR in the in vivo feasibility study is motion sensitivity. RARE‐VTR uses conventional Cartesian k‐space sampling and is more susceptible to respiration‐related motion because of its long acquisition times. Although respiratory triggering can reduce breathing artifacts, it further prolongs the scan. In contrast, PETALUTE uses a non‐Cartesian sampling strategy that is less prone to artifacts from respiration and cardiac motion and provides efficient k‐space coverage for undersampled imaging.^44^ This enables abdominal imaging without respiratory gating within a practical scan time. However, the relative sensitivity of PETALUTE and RARE‐VTR to physiological motion was not systematically evaluated in the present study and should be examined in future work.

This study has several limitations that should be acknowledged. First, PETALUTE images appear slightly blurrier than those acquired with RARE‐VTR (Figure 6). This is primarily because we deliberately used a high undersampling factor together with a CS‐SENSE reconstruction to enable faster, non‐gated whole abdominal T_1_ mapping. For the target nominal resolution (matrix = 256 × 256 × 256, *r* = 128), the Nyquist criterion corresponds to approximately $4\pi \;\times \;r^{2}$ petals (4π×1282≈2.1×105). In comparison, our protocol uses 36,864, which is 18% of the Nyquist sampling density. This level of undersampling reduces image sharpness and fine edge detail, leading to some loss of anatomic definition relative to RARE‐VTR. However, previous work has shown that PETALUTE $T_{1}$ estimates remain stable even at higher undersampling level (18,192 petals) reconstructed with the same matrix size (256 × 256 × 256), so substantial degradation of $T_{1}$ values is not expected at the sampling density used here.^45^ If higher anatomical clarity is desired, the protocol can be adapted by increasing the sampling density.

Second, although the PETALUTE T_1_ mapping approach extends the reliable linear range of ferumoxytol concentrations, it remains limited at very high concentrations. At 5000 µg/mL, the susceptibility of ferumoxytol at 7T results in a local frequency shift of approximately 20 kHz and an estimated T_2_* of about 0.01 ms.^24^ These large off‐resonance effects and extremely short T_2_* lead to considerable signal attenuation and k‐space misregistration that severely degrade $T_{1}$ mapping accuracy. Under these conditions, the current rosette k‐space sampling and reconstruction framework is no longer sufficient to preserve reliable T_1_ information, and additional off‐resonance correction would likely be required. Therefore, 5000 µg/mL is reported here as an upper practical limit of the PETALUTE $T_{1}$ mapping approach for ferumoxytol assessment.

Finally, the in vivo experiments should be interpreted as an initial feasibility demonstration and proof‐of‐concept rather than a comprehensive method validation, given the small sample size. In addition, differences in tumor volume among the studied mice may have influenced the observed imaging features and biological interpretation. Future studies with larger animal cohorts are needed to statistically evaluate measurement accuracy and biological variability in ferumoxytol uptake using PETALUTE $T_{1}$ mapping.

Clinical translation of the present 7T results will require additional validation at 3T and 1.5T, since field strength affects relaxivity, susceptibility behavior, and T_1_ mapping performance.^18^ Accordingly, the present T_1_ mapping protocol will need to be re‐established for each clinical field strength rather than assumed to transfer directly from 7T. In addition, ferumoxytol clinical translation will require careful dose optimization, monitored administration, and safety studies. A reasonable clinic migration pathway would therefore be field‐specific calibration and reproducibility testing at 3T and 1.5T, followed by prospective clinical feasibility studies.

We also note that the present study did not include a dedicated quantitative T_2_* analysis, simulation study, or multi‐echo relaxation modeling to directly disentangle the T_1_ and T_2_* contributions in SPION‐rich regions. Further evaluation of PETALUTE's performance will be carried out using simulations to understand how varying SPION concentration, off‐resonance, and T_2_* shortening affect $T_{1}$ mapping precision. Future studies will also focus on validating in vivo findings with histological evidence using both T_1_ and T_2_* mapping.

## 5 CONCLUSION

In summary, the PETALUTE $T_{1}$ mapping protocol provides more reliable ferumoxytol assessment compared with RARE‐VTR. Uniquely, this protocol enables high‐resolution, non‐gated whole‐abdominal images and dual‐echo imaging within a single scan. This multimodal capability makes PETALUTE particularly valuable for monitoring treatment with SPION‐based therapies.

## FUNDING INFORMATION

Support was provided by Eli Lilly and Company. The authors also acknowledge support from NIH grant P30 CA023168 (Purdue University Institute for Cancer Research) and from Wellcome Trust Collaborative Award 223131/Z/21/Z (UE and SW).

## CONFLICT OF INTEREST STATEMENT

The authors declare no conflicts of interest.

## Supporting information



Supporting Information

## Data Availability

The data that support the findings of this study are available from the corresponding author upon reasonable request.
