# Supplementary material for: Fast and robust T1 mapping based on a 3D dual‐echo UTE sequence (PETALUTE) for superparamagnetic iron oxide nanoparticle (SPION) biodistribution assessment
Source: Med Phys. 2026 Sep 24;53(10):e70675. doi: 10.1002/mp.70675 (PMC13603949; doi:10.1002/mp.70675)
Supplement: Supplementary file 1 [file MP-53-0-s001.docx]

**Supplementary Methods**

**S1 Image quality analysis**

**S1.1 Region-of-interest (ROI) definition**

ROIs were manually drawn in 3D Slicer within the agarose and ferumoxytol compartments of each phantom. The agarose ROI volume was approximately 8.54 mm³, and the ferumoxytol ROI volume was approximately 0.36 mm³. To estimate background noise, noise ROIs were placed in background regions surrounding each phantom. Each noise ROI was matched to the agarose ROI volume (8.54 mm³). A representative example of ROI placement is shown in Figure S1.

All ROI masks were first defined on the PETALUTE images and then resampled to the RARE-VTR image space using the resampling function in 3D Slicer.

**Figure S1.** Representative ROI placement for image-quality analysis on the PETALUTE image of 250 μg/mL phantom. (a) agarose (purple) and four background noise ROIs. (b) ferumoxytol (green) and background noise ROIs.

**S1.2 Signal-to-noise ratio (SNR) and contrast-to-noise ratio (CNR) calculation**

SNR was calculated separately for agarose and ferumoxytol ROIs as:

$$\text{SNR}\text{ }\text{=}\text{ }\text{μ}_{\text{signal}}\text{/}{\bar{\text{σ}}}_{\text{noise}}$$

Equation 1

Where

$${\bar{\text{σ}}}_{\text{noise}}\text{ }\text{=}\text{ }\text{(}\text{σ}_{\text{noise1}}\text{+}\text{σ}_{\text{noise2}}\text{+}\text{σ}_{\text{noise3}}\text{+}\text{σ}_{\text{noise4}}\text{)/4}$$

Equation 2

Ferumoxytol-to-agarose CNR was calculated as:

$$\text{CNR}\text{ }\text{=}\text{ }\text{(}\text{μ}_{\text{ferumoxytol}}\text{-}\text{μ}_{\text{agarose}}\text{)/}{\bar{\text{σ}}}_{\text{noise}}$$

Equation 3

PETALUTE images reconstructed both with and without compressed sensing were evaluated for comparison of SNR and ferumoxytol-to-agarose CNR against RARE-VTR. The reconstruction without compressed sensing was included to allow assessment of PETALUTE image quality without the contribution from compressed-sensing reconstruction. For RARE-VTR, the first echo acquired at TR = 934.58 ms was selected for comparison.

**S2 PETALUTE R_1_ calibration analysis**

PETALUTE mean T_1_ values were extracted from the ferumoxytol ROI defined in Section S1.1 and converted to R_1_ (R_1_=1/T_1_). PETALUTE R_1_ values were plotted against ferumoxytol concentration. Linear regression was performed over the 0-1250 μg/mL range, and the 5000 μg/mL measurement was plotted on the same graph but was not included in the linear fit.

**S3 T_1_ agreement analysis**

For T_1_ agreement analysis, the PETALUTE T_1_ map was resampled to match the spatial resolution and image space of the RARE-VTR T_1_ map, so that the two methods were compared on a common voxel grid. Agreement between PETALUTE with and without B_1_^+^ correction and RARE-VTR T_1_ values was then assessed using Bland–Altman analysis. T_1_ values were extracted from the ROI defined in Section S1.1 in the 0 μg/mL phantom (agarose only).

**Supplementary Results and Discussion**

**S4 Image quality comparison between PETALUTE and RARE-VTR**

Table S1 summarizes agarose SNR for PETALUTE images reconstructed with and without compressed sensing and for RARE-VTR across phantoms with different ferumoxytol concentrations. Table S2 summarizes ferumoxytol SNR, and Table S3 summarizes ferumoxytol-to-agarose CNR.

Overall, RARE-VTR showed higher agarose SNR than PETALUTE across all phantoms (Figure S2a). In contrast, both PETALUTE reconstructions showed higher ferumoxytol SNR than RARE-VTR (Figure S2b). PETALUTE also produced positive ferumoxytol-to-agarose CNR across all phantoms, whereas RARE-VTR did not (Figure S2c). These differences likely reflect the distinct acquisition design of the two methods, including echo time, repetition time, flip angle, sampling strategy, and reconstruction. As a result, PETALUTE and RARE-VTR are not expected to provide equivalent signal weighting or identical sensitivity to ferumoxytol-related contrast, and the SNR and CNR values reported here should therefore be interpreted as descriptive image-quality measures rather than as a basis for direct statistical comparison between methods.

Compressed-sensing reconstruction increased agarose SNR, ferumoxytol SNR, and ferumoxytol-to-agarose CNR in the PETALUTE images (Figure S2). This finding indicates that compressed sensing improved the practical image-quality metrics of PETALUTE images. However, because compressed sensing changes the reconstruction process and may affect noise behavior, these improvements should be interpreted as reconstruction-associated gains within the PETALUTE T_1_ mapping pipeline rather than as purely acquisition-driven differences.

Within the PETALUTE data, ferumoxytol-to-agarose CNR at 5000 μg/mL decreased by 67% relative to 1250 μg/mL with compressed-sensing reconstruction. A 42% decline at 5000 μg/mL relative to 1250 μg/mL was also observed in PETALUTE without compressed-sensing reconstruction. This reduction suggests loss of ferumoxytol contrast at 5000 μg/mL, which could limit the accuracy and reliability of the current PETALUTE T_1_ mapping protocol in that range (Figure S3). Possible contributors include severe susceptibility-related dephasing and T_2_* shortening at extreme ferumoxytol concentrations. However, this interpretation remains tentative because the present study does not directly evaluate the mechanism underlying the observed reduction in contrast.

**Figure S2.** Visual summary of image quality metrics across ferumoxytol concentrations. (a) Agarose signal-to-noise ratio (SNR), (b) ferumoxytol SNR, and (c) ferumoxytol-to-agarose contrast-to-noise ratio (CNR) for PETALUTE reconstructed with compressed sensing (CS), PETALUTE reconstructed without CS, and RARE-VTR.

***Figure S3.*** *PETALUTE R1 calibration curve. A linear regression model was fit over the 0-1250 μm/mL range. The 5000 μm/mL data excluded from the fit and is shown separately for comparison with the fitted trend. Error bars represent the standard deviation.*

**S5 PETALUTE R_1_ calibration curve across full concentration**

Figure S3 shows the PETALUTE R_1_ calibration curve as a function of ferumoxytol concentration. R_1_ increased approximately linearly from 0 to 1250 μg/mL, whereas the 5000 μg/mL mean R_1_ deviated substantially from the fitted trend. This deviation supports the interpretation that 5000 μg/mL falls outside the reliable range of the current PETALUTE T_1_ mapping protocol.

**S6 T_1_ comparison between PETALUTE and RARE-VTR**

Figure S4 shows Bland–Altman comparison between RARE-VTR and PETALUTE T_1_ estimates with and without B_1_^+^ correction. Without B_1_^+^ correction, PETALUTE showed substantial systematic bias relative to RARE-VTR, with a mean bias of -1456.37 ms. After B_1_^+^ correction, the absolute bias was reduced to 562.46 ms, indicating that B_1_^+^ correction reduced the difference between PETALUTE and RARE-VTR T_1_ values. The residual bias more likely reflects the differences in sequence physics rather than partial-volume effects, since the comparison was performed on T_1_ maps at the same spatial resolution. This interpretation is consistent with prior phantom work showing that variable-flip-angle T_1_ measurements exhibit greater bias than recovery-based reference methods and that B_1_^+^ inhomogeneity is a major source of error^1^.

**Figure S4.** Bland–Altman plots comparing PETALUTE- and RARE-VTR T_1_ values. (a) PETALUTE without B_1_^+^ correction versus RARE-VTR. (b) PETALUTE with B_1_^+^ correction versus RARE-VTR. The solid red line indicates the mean bias, and the dashed black lines indicate the 95% limits of agreement.

**Reference**

1.	Keenan KE, Gimbutas Z, Dienstfrey A, et al. Multi-site, multi-platform comparison of MRI T_1_ measurement using the system phantom. *PLOS ONE*. 2021;16(6):e0252966. doi:10.1371/journal.pone.0252966

**Supplementary Tables**

**Table S1.** Agarose signal-to-noise ratio (SNR) of PETALUTE with and without compressed sensing and of RARE-VTR for each phantom.

| **Ferumoxytol concentration** | **PETALUTE (without CS_a_)** | **PETALUTE**  **(with CS)** | **RARE-VTR** |
| --- | --- | --- | --- |
| 0 µg/mL | 32 | 69 | 131 |
| 100 µg/mL | 34 | 69 | 120 |
| 250 µg/mL | 37 | 87 | 116 |
| 750 µg/mL | 33 | 74 | 94 |
| 1000 µg/mL | 34 | 72 | 112 |
| 1250 µg/mL | 35 | 89 | 99 |
| 5000 µg/mL | 34 | 67 | 90 |

_a_ CS = Compressed sensing

**Table S2.** Ferumoxytol signal-to-noise ratio (SNR) of PETALUTE with and without compressed sensing and of RARE-VTR for each phantom.

| **Ferumoxytol concentration** | **PETALUTE (without CS_a_)** | **PETALUTE**  **(with CS)** | **RARE-VTR** |
| --- | --- | --- | --- |
| 0 µg/mL | 86 | 164 | 51 |
| 100 µg/mL | 116 | 248 | 3 |
| 250 µg/mL | 97 | 226 | 3 |
| 750 µg/mL | 112 | 214 | 4 |
| 1000 µg/mL | 101 | 274 | 3 |
| 1250 µg/mL | 71 | 128 | 7 |

_a_ CS = Compressed sensing

**Table S3.** Ferumoxytol-to-agarose contrast-to-noise ratio (CNR) of PETALUTE with and without compressed sensing and of RARE-VTR for each phantom.

| **Ferumoxytol concentration** | **PETALUTE (without CS_a_)** | **PETALUTE**  **(with CS)** | **RARE-VTR** |
| --- | --- | --- | --- |
| 0 µg/mL | 51 | 95 | -68 |
| 100 µg/mL | 79 | 161 | -112 |
| 250 µg/mL | 64 | 153 | -91 |
| 750 µg/mL | 78 | 142 | -109 |
| 1000 µg/mL | 66 | 186 | -95 |
| 1250 µg/mL | 38 | 61 | -84 |

_a_ CS = Compressed sensing
